# Capivasertib and fulvestrant for patients with HR-positive/HER2-negative advanced breast cancer: analysis of the subgroup of patients from Japan in the phase 3 CAPItello-291 trial

**DOI:** 10.1007/s12282-024-01640-z

**Published:** 2024-10-08

**Authors:** Eriko Tokunaga, Hiroji Iwata, Mitsuya Itoh, Tetsuhiko Taira, Tatsuya Toyama, Toshiro Mizuno, Akihiko Osaki, Yasuhiro Yanagita, Seigo Nakamura, Rikiya Nakamura, Tomoko Sambe, Toshiaki Ozaki, Gaia Schiavon, Sacha J. Howell, Masakazu Toi

**Affiliations:** 1 National Hospital Organization (NHO) Kyushu Cancer Center, Fukuoka, Japan; 2 Aichi Cancer Center Hospital, Aichi, Japan; 3 Hiroshima City Hiroshima Citizens Hospital, Hiroshima, Japan; 4 Sagara Hospital, Social Medical Corporation Hakuaikai, Kagoshima, Japan; 5 Nagoya City University Hospital, Aichi, Japan; 6 Mie University Hospital, Mie, Japan; 7 Saitama Medical University International Medical Center, Saitama, Japan; 8 Gunma Prefectural Cancer Center, Gunma, Japan; 9 Showa University Hospital, Tokyo, Japan; 10 Chiba Cancer Center, Chiba, Japan; 11 Oncology R&D, AstraZeneca, Osaka, Japan; 12 Oncology R&D, AstraZeneca, Cambridge, UK; 13 The Christie NHS Foundation Trust, Manchester, UK; 14 Tokyo Metropolitan Cancer and Infectious Disease Center, Komagome Hospital, Tokyo, Japan

**Keywords:** AKT inhibitor, Capivasertib, Hormone receptor-positive breast cancer, Post-CDK4/6

## Abstract

**Background:**

In CAPItello-291, capivasertib–fulvestrant significantly improved progression-free survival (PFS) versus placebo–fulvestrant in the overall and *PIK3CA/AKT1/PTEN*-altered population with hormone receptor-positive (HR-positive)/human epidermal growth factor receptor 2-negative (HER2-negative) advanced breast cancer. Capivasertib–fulvestrant is approved in Japan for the treatment of patients with one or more tumor biomarker alterations (*PIK3CA*, *AKT1* or *PTEN*). Here, we report outcomes in the CAPItello-291 subgroup of patients from Japan.

**Methods:**

Adults with HR-positive/HER2-negative advanced breast cancer whose disease had relapsed or progressed during or after treatment with an aromatase inhibitor, with or without previous cyclin-dependent kinase 4/6 (CDK4/6) inhibitor therapy, were randomly assigned (1:1 ratio) to receive capivasertib or placebo, plus fulvestrant. The dual primary endpoint was investigator-assessed PFS in the overall and *PIK3CA/AKT1/PTEN*-altered population. Safety was a secondary endpoint.

**Results:**

Of 708 patients randomized in CAPItello-291, 78 were from Japan (37 randomized to capivasertib–fulvestrant and 41 to placebo–fulvestrant). In the Japan subgroup, PFS numerically favored the capivasertib–fulvestrant arm (hazard ratio 0.73; 95% CI 0.40–1.28), consistent with the analysis of PFS in the global population. Similarly, in the Japan subgroup of patients with *PIK3CA/AKT1/PTEN*–altered tumors, PFS favored the capivasertib–fulvestrant arm (hazard ratio 0.65; 95% CI 0.29–1.39), consistent with the global population. The adverse event profile of capivasertib–fulvestrant in the Japan subgroup was broadly similar to that in the global population; no new safety concerns were identified.

**Conclusion:**

Outcomes in the Japan subgroup were broadly similar to those of the global population, supporting the clinical benefit of capivasertib–fulvestrant in treating HR-positive/HER2-negative advanced breast cancer that has progressed on, or after, an endocrine-based regimen.

**Supplementary Information:**

The online version contains supplementary material available at 10.1007/s12282-024-01640-z.

## Introduction

Breast cancer is the most common cancer among women in Japan, and the third leading cause of cancer-related death overall [1]. Furthermore, although breast cancer incidence rates have been historically low in Japan, they are now rising rapidly [2, 3]. Approximately 70% of breast cancers in Japan are hormone receptor-positive (HR-positive)/human epidermal growth factor receptor 2-negative (HER2-negative) [4].

Endocrine therapy, often an aromatase inhibitor (AI), in combination with a cyclin-dependent kinase 4/6 (CDK4/6) inhibitor is the recommended first-line treatment option for certain patients with HR-positive/HER2-negative advanced breast cancer both internationally [5–7] and in Japan [8]. However, in many patients, the disease eventually develops resistance to endocrine-based treatment regimens, and so subsequent treatment options are needed in combination with endocrine therapy to extend the utility of treatment and defer the need for chemotherapy. One currently recommended treatment approach in patients whose disease develops resistance to an aromatase inhibitor is fulvestrant, a selective estrogen receptor degrader, administered either as monotherapy or as part of combination treatment [5–8].

AKT is the key node of the phosphoinositide 3-kinase (PI3K)/AKT serine/threonine protein kinase (AKT) pathway, a major signaling pathway involved in the regulation of cell metabolism, proliferation and survival [9]. Overactivation of the PI3K/AKT pathway occurs frequently in HR-positive/HER2-negative advanced breast cancer [9, 10], and is associated with progression on endocrine therapy [10–14]. Capivasertib is a potent, selective inhibitor of AKT1, AKT2, and AKT3 [15]. In the global Phase 3 randomized CAPItello-291 trial in patients with HR-positive advanced breast cancer whose disease had progressed during or after previous AI therapy with or without a CDK4/6 inhibitor, the addition of capivasertib to fulvestrant resulted in statistically significant and clinically meaningful improvement in the dual primary endpoints of progression-free survival (PFS) in the overall population [hazard ratio 0.60; 95% confidence interval (CI) 0.51–0.71; *P* < 0.001] and in patients with *PIK3CA/AKT1/PTEN*-altered tumors (hazard ratio 0.50; 95% CI 0.38–0.65; *P* < 0.001) compared with placebo plus fulvestrant [16]. Frequent AEs associated with PI3K/AKT pathway inhibition (diarrhea, rash, and hyperglycemia) occurred early and were manageable [17], and capivasertib–fulvestrant delayed time to deterioration of global health status/quality of life and maintained other dimensions of HRQOL compared to placebo–fulvestrant [18].

Data from CAPItello-291 have led to the regulatory approval of capivasertib–fulvestrant in patients with HR-positive/HER2-negative advanced breast cancer and one or more tumor biomarker alterations (*PIK3CA*, *AKT1* or *PTEN*) in several countries, including in Japan and the US [19, 20]. Capivasertib–fulvestrant has also been recommended as a treatment option in clinical practice guidelines [6, 7, 21].

Here, we report the efficacy and safety of capivasertib plus fulvestrant in the subgroup of patients who were enrolled in Japan and review the findings in context of the outcomes observed in the global CAPItello-291 population.

## Patients and methods

### Study design

CAPItello-291 (NCT04305496) is a global Phase 3, double-blind, placebo-controlled randomized study. The design of CAPItello-291, including inclusion/exclusion criteria and randomization and stratification factors, has been published in detail previously [16]. Briefly, patients with HR-positive/HER2-negative locally advanced or metastatic breast cancer that had progressed on a prior AI, with or without a CDK4/6 inhibitor, in the metastatic setting or on, or within, 12 months of the end of treatment with a (neo)adjuvant AI were eligible for inclusion in the study. Patients could have received up to two prior lines of endocrine therapy and one prior line of chemotherapy in the advanced setting, and prior AI therapy was not required to be the most recent treatment. Adult patients ≥ 20 years of age in Japan were eligible for study inclusion, ≥ 18 years of age in other regions [16].

### Treatments and assessments

Patients were randomized 1:1 to receive oral capivasertib (400 mg) twice daily (BID) on an intermittent dosing schedule (4 days on, 3 days off) and fulvestrant (500 mg intramuscularly given per standard of care every 14 days for the first three injections, then every 28 days) or matching placebo and fulvestrant. Randomization was stratified by the presence of liver metastases, prior use of CDK4/6 inhibitors and geographic region.

One treatment cycle was defined as 4 weeks of capivasertib or placebo, and treatment continued until disease progression as per Response Evaluation Criteria in Solid Tumors (RECIST) version 1.1, unacceptable toxicity, withdrawal of consent, or death.

Tumor assessments were conducted at screening (within 4 weeks prior to randomization), every 8 weeks for 18 months, and then every 12 weeks until disease progression, using computed tomography or magnetic resonance imaging scans (or both). Radiographic bone scans were performed at screening and repeated as clinically indicated. Patients who discontinued capivasertib or fulvestrant for any reason other than disease progression continued to undergo scans every 8 weeks until evidence of disease progression.

Adverse events (AEs) were reported throughout the study, from the date of informed consent until 30 (+ 7) days after discontinuation of treatment. Any AE that started after the first treatment dose or before the first dose but worsened after the first dose was considered a treatment-emergent AE and included within AE summaries. AE severity was graded using the National Cancer Institute Common Terminology Criteria for Adverse Events (NCI CTCAE) v5.0. An independent data monitoring committee assessed the progress of CAPItello-291 approximately every 6 months and reviewed unblinded safety data.

### Outcomes

The dual primary endpoints of CAPItello-291 were investigator-assessed PFS in the global CAPItello-291 population and in the subset of patients with *PIK3CA/AKT1/PTEN*–altered tumors. Overall survival and objective response rate (ORR), as well as safety, were secondary endpoints.

### Statistical analysis

A detailed description of the statistical analysis of CAPItello-291, including sample size considerations and statistical power, has been published in detail previously [16]. Analysis of the Japan subgroup was performed using the global CAPItello-291 primary analysis data with a cut-off date of August 15, 2022. The primary analysis was conducted when 551 events of disease progression or death had occurred in the global CAPItello-291 population and the results have been reported previously [16]. All statistical analyses are considered exploratory in the Japan subgroup, and comparison statistics were performed only if sufficient numbers of events or patients were available (e.g., ≥ 20 PFS or overall survival events across both treatment arms).

All patients in the Japan subgroup who were randomized were included in the analysis of efficacy. Analysis was conducted based on the predefined pooling strategy. Randomization stratification factors were dropped per individual endpoint in the following order: geographic region, presence or absence of liver metastases, and previous CDK4/6 inhibitor use. Time-to-event analyses were conducted using Kaplan–Meier methodology, and hazard ratios were estimated using the Cox proportional hazard model. Analysis of objective response was conducted in patients with measurable disease at baseline and performed using the Cochran–Mantel–Haenszel test.

All patients who received at least one dose of capivasertib, fulvestrant or placebo were included in the safety analysis, and safety and tolerability data were summarized using descriptive statistics, comprising the number and percentage of patients reporting each AE. The group term of rash was analyzed retrospectively and comprised the preferred terms of rash, rash macular, rash maculopapular, rash papular, and rash pruritic.

All data reported are descriptive; CAPItello-291 was not powered to detect treatment differences in the Japan subgroup.

## Results

### Patient disposition

As described previously, 901 patients were enrolled in CAPItello-291 between June 2, 2020 and October 13, 2021, and 708 patients were randomized to receive either capivasertib–fulvestrant (*n* = 355) or placebo–fulvestrant (*n* = 353) [16]. Of these, 87 patients from Japan were enrolled between June 2, 2020 and September 14, 2021, 78 of whom were randomized to receive either capivasertib–fulvestrant (*n* = 37) or placebo–fulvestrant (*n* = 41) (Supplemental Fig. 1). All 78 patients randomized to treatment went on to receive treatment, with a median duration of treatment of 7.5 months for capivasertib and 12.1 months for fulvestrant in the capivasertib–fulvestrant arm, and 9.1 months for placebo and 9.2 months for fulvestrant in the placebo–fulvestrant arm.

At the data cut-off, 13 patients (35.1%) from Japan were continuing to be treated with capivasertib and 11 patients (26.8%) were continuing to be treated with placebo. Capivasertib was discontinued in 24 patients (64.9%), and placebo was discontinued in 30 patients (73.2%). The main reason for discontinuation of capivasertib or placebo was radiological disease progression (Supplemental Fig. 1).

### Baseline demographics and disease characteristics

All patients from Japan were female, with a median age of 61 years, and approximately three-quarters were post-menopausal. Demographics and disease characteristics were generally balanced between treatment arms in patients from Japan and most were similar between the Japan subgroup and the global CAPItello-291 population (Table 1). However, it should be noted that more patients from Japan (89.7%) had an Eastern Cooperative Oncology Group Performance Status (ECOG PS) score of 0 compared with the global CAPItello-291 population (65.7%), as well as ER-positive plus progesterone receptor (PgR)-positive status (80.8% versus 70.8%), and fewer patients from Japan (30.8%) had the presence of liver metastases at baseline compared with the global CAPItello-291 population (43.2%). In terms of prior therapy, fewer patients from Japan had been treated with prior endocrine therapy for advanced breast cancer (65.4%) or CDK4/6 inhibitors (16.7%) compared with the global CAPItello-291 population (86.7% and 70.1%, respectively); as such, more patients in Japan received study treatment as their first-line treatment. The most common reason for patients in Japan not previously having received a CDK4/6 inhibitor was due to patient or health care provider preference (83.1%).

**Table 1:** Baseline demographics and disease characteristics

	All patients				Patients with *PIK3CA/AKT1/PTEN*-altered tumors			
	Japan subgroup		Global CAPItello-291 population		Japan subgroup		Global CAPItello-291 population	
	Capivasertib–fulvestrant (*n* = 37)	Placebo–fulvestrant (*n* = 41)	Capivasertib–fulvestrant (*n* = 355)	Placebo–fulvestrant (*n* = 353)	Capivasertib–fulvestrant (n = 19)	Placebo–fulvestrant (*n* = 19)	Capivasertib–fulvestrant (*n* = 155)	Placebo–fulvestrant (*n* = 134)
*Demographics*								
Median age (range), years	61 (42–84)	61 (37–83)	59 (26–84)	58 (26–90)	61 (48–84)	62 (39–80)	58 (36–84)	60 (34–90)
Female sex, *n* (%)	37 (100)	41 (100)	352 (99.2)	349 (98.9)	19 (100)	19 (100)	153 (98.7)	134 (100)
Postmenopausal, *n* (%)	29 (78.4)	29 (70.7)	287 (80.8)	260 (73.7)	15 (78.9)	14 (73.7)	130 (83.9)	105 (78.4)
Asian, *n* (%)	37 (100)	41 (100)	95 (26.8)	94 (26.6)	19 (100)	19 (100)	48 (31.0)	35 (26.1)
*ECOG performance status score, n (%)*								
0	35 (94.6)	35 (85.4)	224 (63.1)	241 (68.3)	19 (100)	18 (94.7)	93 (60.0)	97 (72.4)
1	2 (5.4)	6 (14.6)	131 (36.9)	111 (31.4)	0	1 (5.3)	62 (40.0)	36 (26.9)
2	0	0	0	1 (0.3)	0	0	0	1 (0.7)
*Disease characteristics*								
Liver metastasis,^a^ *n* (%)	11 (29.7)	13 (31.8)	156 (43.9)	150 (42.5)	4 (21.1)	6 (31.6)	70 (45.2)	53 (39.6)
*Number of previous therapies for advanced breast cancer, n (%)*								
0	11 (29.7)	16 (39.0)	37 (10.4)	52 (14.7)	5 (26.3)	7 (36.8)	12 (7.7)	20 (14.9)
1	17 (45.9)	16 (39.0)	235 (66.2)	208 (58.9)	10 (52.6)	6 (31.6)	107 (69.0)	79 (59.0)
2	8 (21.6)	7 (17.1)	73 (20.6)	77 (21.8)	4 (21.1)	4 (21.1)	31 (20.0)	29 (21.6)
3	1 (2.7)	2 (4.9)	10 (2.8)	16 (4.5)	0	2 (10.5)	5 (3.2)	6 (4.5)
*Hormone-receptor status,*^*b*^* n (%)*								
ER-positive, PgR-positive	30 (81.1)	33 (80.5)	255 (71.8)	246 (69.7)	16 (84.2)	14 (73.7)	116 (74.8)	101 (75.4)
ER-positive, PgR-negative	7 (18.9)	8 (19.5)	94 (26.5)	103 (29.2)	3 (15.8)	5 (26.3)	35 (22.6)	31 (23.1)
ER-positive, with unknown PgR status	0	0	5 (1.4)	4 (1.1)	0	0	4 (2.6)	2 (1.5)
*Endocrine status,*^*c*^* n (%)*								
Primary resistance	15 (40.5)	14 (34.1)	127 (35.8)	135 (38.2)	10 (52.6)	6 (31.6)	60 (38.7)	55 (41.0)
Secondary resistance	22 (59.5)	27 (65.9)	228 (64.2)	218 (61.8)	9 (47.4)	13 (68.4)	95 (61.3)	79 (59.0)
*Number of previous endocrine therapies for advanced breast cancer, n (%)*								
0	11 (29.7)	16 (39.0)	39 (11.0)	54 (15.3)	5 (26.3)	7 (36.8)	13 (8.4)	20 (14.9)
1	19 (51.4)	18 (43.9)	287 (80.8)	252 (71.4)	11 (57.9)	8 (42.1)	131 (84.5)	96 (71.6)
2	7 (18.9)	7 (17.1)	29 (8.2)	47 (13.3)	3 (15.8)	4 (21.1)	11 (7.1)	18 (13.4)
*Previous CDK4/6 inhibitor, n (%)*								
As neoadjuvant or adjuvant therapy	0	0	2 (0.6)	5 (1.4)	0	0	0	2 (1.5)
As therapy for advanced breast cancer	5 (13.5)	8 (19.5)	245 (69.0)	244 (69.1)	4 (21.1)	3 (15.8)	113 (72.9)	91 (67.9)
*Previous chemotherapy, n (%)*								
As neoadjuvant or adjuvant therapy	15 (40.5)	19 (46.3)	180 (50.7)	170 (48.2)	6 (31.6)	8 (42.1)	79 (51.0)	67 (50.0)
As therapy for advanced breast cancer	3 (8.1)	4 (9.8)	65 (18.3)	64 (18.1)	1 (5.3)	4 (21.1)	30 (19.4)	23 (17.2)

*AKT1* Akt serine/threonine kinase 1, *CDK* 4/6 cyclin-dependent kinases 4/6, *ECOG* Eastern Cooperative Oncology Group, *ER* estrogen receptor, *PIK3CA* catalytic subunit of phosphatidylinositol-3-kinase, *PgR* progesterone receptor, *PTEN* phosphatase and tensin homolog

^a^Baseline stratification factor

^b^One patient in the global CAPItello-291 population treated with capivasertib–fulvestrant was ER-negative

^c^Primary and secondary resistance were defined using the 4th European School of Oncology-European Society for Medical Oncology International Consensus Guidelines for advanced breast cancer

In total, 38 patients from Japan had *PIK3CA/AKT1/PTEN*-altered tumors (48.7%), 19 in each treatment arm, and a higher proportion compared with the global CAPItello-291 population (40.8%) (Supplemental Table 1). Patients from Japan with *PIK3CA/AKT1/PTEN*-altered tumors had similar demographics and disease characteristics to the overall Japan subgroup (Table 1).

### Efficacy

**Fig. 1:**
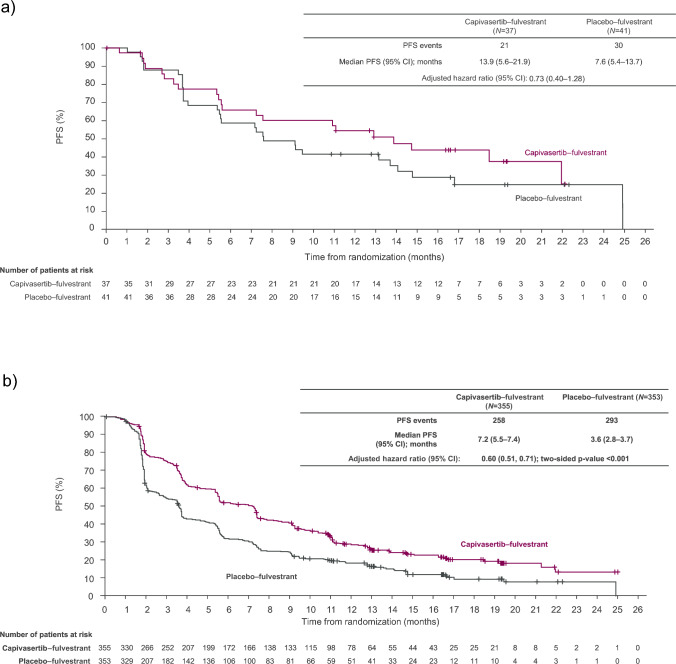
Investigator-assessed PFS in **a** the Japan subgroup, and **b** the global CAPItello-291 population. The hazard ratio was estimated in the Japan subgroup using the Cox proportional hazards model stratified by previous CDK4/6 inhibitor use, and in the global CAPItello-291 population using the Cox proportional hazards model stratified according to the presence or absence of liver metastases, previous CDK4/6 inhibitor use, and geographic region. Tick marks indicate censored data. **b** From *New England Journal of Medicine*. Turner et al. [16]. Copyright © (2023) Massachusetts Medical Society. Reprinted with permission. *CI* confidence interval, *PFS* progression-free survival

As of August 15, 2022, the median duration of follow-up for investigator-assessed PFS in censored patients in the overall population of the Japan subgroup was 16.5 months (range 0.0–22.1) in the capivasertib–fulvestrant arm, and 16.6 months (range 10.8–22.3) in the placebo–fulvestrant arm. In the Japan subgroup, investigator-assessed median PFS was 13.9 months in the capivasertib–fulvestrant arm and 7.6 months in the placebo–fulvestrant arm (hazard ratio 0.73; 95% CI 0.40–1.28) (Fig. 1a). The addition of capivasertib to fulvestrant led to relative improvement in PFS in the Japan subgroup as was also observed for PFS in the global CAPItello-291 population (Fig. 1b; hazard ratio 0.60; 95% CI 0.51–0.71) [16].

**Fig. 2:**
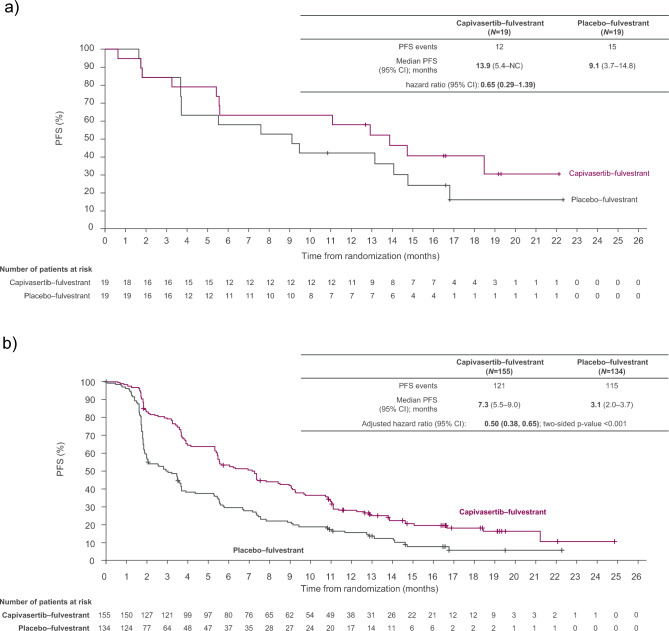
Investigator-assessed PFS in patients with *PIK3CA/AKT1/PTEN*-altered tumors in **a** the Japan subgroup, and **b** the global CAPItello-291 population. The hazard ratio was estimated in the Japan subgroup using an unstratified Cox proportional hazards model, and in the global CAPItello-291 population using the Cox proportional hazards model stratified according to the presence or absence of liver metastases and previous CDK4/6 inhibitor use. Tick marks indicate censored data. **b** *New England Journal of Medicine*. Turner et al. [16]. Copyright © (2023) Massachusetts Medical Society. Reprinted with permission. *AKT1* Akt serine/threonine kinase 1, *CI* confidence interval, *NC* not calculable, *PFS* progression-free survival, *PIK3CA* catalytic subunit of phosphatidylinositol-3-kinase, *PTEN* phosphatase and tensin homolog

In the Japan subgroup of patients with *PIK3CA/AKT1/PTEN*-altered tumors, investigator-assessed median PFS was 13.9 months in the capivasertib–fulvestrant arm and 9.1 months in the placebo–fulvestrant arm (hazard ratio 0.65; 95% CI 0.29–1.39) (Fig. 2a), consistent with findings in the global CAPItello-291 population favoring capivasertib–fulvestrant (Fig. 2b; hazard ratio 0.50; 95% CI 0.38–0.65) [16].

In the Japan subgroup of 40 patients with *PIK3CA/AKT1/PTEN*-non-altered tumors (including patients with no valid next-generation sequencing result), a relative improvement in PFS was also shown with the addition of capivasertib to fulvestrant (Supplemental Fig. 2a; hazard ratio 0.82; 95% CI 0.34–1.86). Exploratory analysis in the same patient population in the global CAPItello-291 population also numerically favored capivasertib–fulvestrant (Supplemental Fig. 2b; hazard ratio 0.70; 95% CI 0.56–0.88) [16]. In the Japan subgroup of 34 patients with confirmed *PIK3CA/AKT1/PTEN*-non-altered tumors (excluding patients with no valid next-generation sequencing result), a PFS benefit was demonstrated with the addition of capivasertib to fulvestrant (hazard ratio 0.69; 95% CI 0.26–1.68), again in line with the exploratory analysis in the same patient population in the global CAPItello-291 population (hazard ratio 0.79; 95% CI 0.61–1.02) [16].

Investigator-assessed ORR favored the capivasertib–fulvestrant arm in the Japan subgroup of patients with measurable disease at baseline, with an odds ratio of 1.48 (95% CI 0.52–4.21), and in the Japan subgroup of patients with *PIK3CA/AKT1/PTEN*-altered tumors and measurable disease at baseline, with an odds ratio of 2.05 (95% CI 0.41–10.24). The observed trend for a higher proportion of patients having an objective response with capivasertib–fulvestrant versus placebo–fulvestrant was consistent with the trend observed in the global CAPItello-291 population (Table 2).

**Table 2:** Tumor response by investigator assessment

	All patients				Patients with *PIK3CA/AKT1/PTEN*-altered tumors			
	Japan subgroup		Global CAPItello-291 population		Japan subgroup		Global CAPItello-291 population	
	Capivasertib–fulvestrant (*n* = 37)	Placebo–fulvestrant (*n* = 41)	Capivasertib–fulvestrant (*n* = 355)	Placebo–fulvestrant (*n* = 353)	Capivasertib–fulvestrant (*n* = 19)	Placebo–fulvestrant (*n* = 19)	Capivasertib–fulvestrant (*n* = 155)	Placebo–fulvestrant (*n* = 134)
Number of patients with measurable disease at baseline, *n*	34	41	310	320	18	19	132	124
Objective response rate, *n* (%)	10 (29.4)	9 (22.0)	71 (22.9)	39 (12.2)	5 (27.8)	3 (15.8)	38 (28.8)	12 (9.7)
Odds ratio (95% CI)	1.48 (0.52–4.21)		2.19 (1.42–3.36)		2.05 (0.41–10.24)		3.93 (1.93–8.04)	
Best overall response, *n*	37	41	355	353	19	19	155	134
Complete response	2 (5.4)	1 (2.4)	4 (1.1)	1 (0.3)	1 (5.3)	0	3 (1.9)	0
Partial response	9 (24.3)	8 (19.5)	68 (19.2)	38 (10.8)	4 (21.1)	3 (15.8)	35 (22.6)	12 (9.0)
Stable disease (≥ 8 weeks)	21 (56.8)	27 (65.9)	187 (52.7)	152 (43.1)	11 (57.9)	13 (68.4)	84 (54.2)	55 (41.0)
Progressive disease	4 (10.8)	5 (12.2)	83 (23.4)	149 (42.2)	3 (15.8)	3 (15.8)	31 (20.0)	62 (46.3)
Non-evaluable	1 (2.7)	0	13 (3.7)	13 (3.7)	0	0	2 (1.3)	5 (3.7)
Clinical benefit rate, *n*/*N* (%)	25/37 (67.6)	25/41 (61.0)	182/355 (51.3)	111/353 (31.4)	13/19 (68.4)	11/19 (57.9)	87/155 (56.1)	37/134 (27.6)
Median duration of response, months (IQR)	10.2 (3.7–20.3)	21.4 (9.1–21.4)	9.8 (5.8–20.3)	8.4 (5.0–17.6)	NC (10.2–NC)	9.2 (9.1–NC)	9.4 (6.1–19.5)	8.6 (5.0–9.2)

*AKT1* Akt serine/threonine kinase 1, *CI* confidence interval, *IQR* interquartile range, *NC* not calculable, *PIK3CA* catalytic subunit of phosphatidylinositol-3-kinase, *PTEN* phosphatase and tensin homolog, *RECIST* Response Evaluation Criteria in Solid Tumors

At the time of analysis, an insufficient number of events (< 20 across treatment groups) for a formal analysis of overall survival had occurred in the Japan subgroup (6 deaths in the capivasertib–fulvestrant arm and 5 deaths in the placebo–fulvestrant arm).

### Safety

All 78 randomized patients in the Japan subgroup received treatment in CAPItello-291 and so all were assessed for safety (37 patients in the capivasertib–fulvestrant arm and 41 patients in the placebo–fulvestrant arm). In the Japan subgroup, AEs of any grade were reported in all 37 patients (100%) in the capivasertib–fulvestrant arm and in 34 patients (82.9%) in the placebo–fulvestrant arm, similar any-grade AE rates to those observed in the global CAPItello-291 population (Table 3).

**Table 3:** Most frequent AEs with capivasertib–fulvestrant (≥ 10% incidence in the Japan subgroup)

	Japan subgroup										Global CAPItello-291 population									
AE, *n*, (%)	Capivasertib–fulvestrant (*n* = 37)					Placebo–fulvestrant (*n* = 41)					Capivasertib–fulvestrant (*n* = 355)					Placebo–fulvestrant (*n* = 350)				
Grade	Any	1	2	3	4	Any	1	2	3	4	Any	1	2	3	4	Any	1	2	3	4
Any AE	37 (100)	4 (10.8)	13 (35.1)	19 (51.4)	1 (2.7)	34 (82.9)	15 (36.6)	13 (31.7)	4 (9.8)	2 (4.9)	343 (96.6)	52 (14.6)	139 (39.2)	139 (39.2)	9 (2.5)	288 (82.3)	115 (32.9)	118 (33.7)	44 (12.6)	10 (2.9)
Diarrhea	27 (73.0)	19 (51.4)	4 (10.8)	4 (10.8)	0	9 (22.0)	6 (14.6)	3 (7.3)	0	0	257 (72.4)	164 (46.2)	60 (16.9)	33 (9.3)	0	70 (20.0)	60 (17.1)	9 (2.6)	1 (0.3)	0
Rash^a^	18 (48.6)	5 (13.5)	6 (16.2)	7 (18.9)	0	3 (7.3)	3 (7.3)	0	0	0	135 (38.0)	57 (16.1)	35 (9.9)	43 (12.1)	0	25 (7.1)	19 (5.4)	5 (1.4)	1 (0.3)	0
Stomatitis	11 (29.7)	4 (10.8)	7 (18.9)	0	0	6 (14.6)	4 (9.8)	2 (4.9)	0	0	52 (14.6)	24 (6.8)	21 (5.9)	7 (2.0)	0	17 (4.9)	15 (4.3)	2 (0.6)	0	0
Pyrexia	9 (24.3)	5 (13.5)	4 (10.8)	0	0	5 (12.2)	5 (12.2)	0	0	0	32 (9.0)	19 (5.4)	12 (3.4)	1 (0.3)	0	14 (4.0)	12 (3.4)	2 (0.6)	0	0
Vomiting	9 (24.3)	8 (21.6)	1 (2.7)	0	0	1 (2.4)	1 (2.4)	0	0	0	73 (20.6)	54 (15.2)	13 (3.7)	6 (1.7)	0	17 (4.9)	10 (2.9)	5 (1.4)	2 (0.6)	0
Nausea	8 (21.6)	8 (21.6)	0	0	0	5 (12.2)	3 (7.3)	2 (4.9)	0	0	123 (34.6)	85 (23.9)	35 (9.9)	3 (0.8)	0	54 (15.4)	42 (12.0)	10 (2.9)	2 (0.6)	0
Dry skin	7 (18.9)	4 (10.8)	3 (8.1)	0	0	3 (7.3)	2 (4.9)	1 (2.4)	0	0	25 (7.0)	20 (5.6)	5 (1.4)	0	0	15 (4.3)	13 (3.7)	1 (0.3)	1 (0.3)	0
Headache	7 (18.9)	6 (16.2)	1 (2.7)	0	0	7 (17.1)	7 (17.1)	0	0	0	60 (16.9)	47 (13.2)	12 (3.4)	1 (0.3)	0	43 (12.3)	33 (9.4)	8 (2.3)	2 (0.6)	0
Cystitis	6 (16.2)	0	6 (16.2)	0	0	1 (2.4)	0	1 (2.4)	0	0	12 (3.4)	3 (0.8)	8 (2.3)	1 (0.3)	0	1 (0.3)	0	1 (0.3)	0	0
Hyperglycemia	6 (16.2)	2 (5.4)	3 (8.1)	1 (2.7)	0	2 (4.9)	2 (4.9)	0	0	0	58 (16.3)	24 (6.8)	26 (7.3)	7 (2.0)	1 (0.3)	13 (3.7)	8 (2.3)	4 (1.1)	1 (0.3)	0
Malaise	6 (16.2)	4 (10.8)	2 (5.4)	0	0	6 (14.6)	4 (9.8)	2 (4.9)	0	0	10 (2.8)	7 (2.0)	3 (0.8)	0	0	7 (2.0)	5 (1.4)	2 (0.6)	0	0
Insomnia	5 (13.5)	4 (10.8)	1 (2.7)	0	0	1 (2.4)	1 (2.4)	0	0	0	22 (6.2)	15 (4.2)	7 (2.0)	0	0	21 (6.0)	17 (4.9)	4 (1.1)	0	0
Pruritus	5 (13.5)	3 (8.1)	2 (5.4)	0	0	4 (9.8)	2 (4.9)	2 (4.9)	0	0	44 (12.4)	32 (9.0)	10 (2.8)	2 (0.6)	0	23 (6.6)	19 (5.4)	4 (1.1)	0	0
Back pain	4 (10.8)	4 (10.8)	0	0	0	2 (4.9)	2 (4.9)	0	0	0	32 (9.0)	17 (4.8)	14 (3.9)	1 (0.3)	0	24 (6.9)	14 (4.0)	7 (2.0)	3 (0.9)	0
Drug eruption	4 (10.8)	0	0	4 (10.8)	0	0	0	0	0	0	4 (1.1)	0	0	4 (1.1)	0	0	0	0	0	0
Dysgeusia	4 (10.8)	4 (10.8)	0	0	0	1 (2.4)	1 (2.4)	0	0	0	21 (5.9)	17 (4.8)	4 (1.1)	0	0	4 (1.1)	4 (1.1)	0	0	0

*AE* adverse event

^a^Group term (preferred terms): rash (rash, rash macular, maculopapular rash, rash papular, rash pruritic)

The AE profile of capivasertib–fulvestrant in the Japan subgroup was broadly consistent with that in the global CAPItello-291 population (Table 3). In the Japan subgroup, the most frequent AEs of any grade (≥ 25% of patients in the capivasertib–fulvestrant arm) were diarrhea (73.0% of patients versus 22.0% patients in the placebo–fulvestrant arm), rash (group term; 48.6% of patients versus 7.3% patients in the placebo–fulvestrant arm), and stomatitis (29.7% of patients versus 14.6% patients in the placebo–fulvestrant arm), and the most frequent AEs of grade 3 (≥ 10% of patients in the capivasertib–fulvestrant arm) were rash (group term; 18.9% of patients versus no patients in the placebo–fulvestrant arm), and diarrhea and drug eruption (both 10.8% versus 0%, respectively). Serious AEs were reported in 5 patients (13.5%) in the capivasertib–fulvestrant arm and in 2 patients (4.9%) in the placebo–fulvestrant arm in the Japan subgroup (Supplemental Table 2). There were no deaths reported due to AEs.

AEs leading to a dose interruption occurred in 21 patients (56.8%) receiving capivasertib in the Japan subgroup, compared with 5 (12.2%) receiving placebo, and AEs leading to dose reduction occurred in 10 patients (27.0%) receiving capivasertib in the Japan subgroup, compared with 2 (4.9%) receiving placebo. Discontinuation due to AEs occurred in 9 patients (24.3%) receiving capivasertib–fulvestrant in the Japan subgroup, and in no patients receiving placebo–fulvestrant. The proportions of patients experiencing AEs leading to discontinuation or dose modification were higher in the Japan subgroup than in the global CAPItello-291 population (Supplemental Table 2). Seven patients (18.9%) receiving capivasertib–fulvestrant in the Japan subgroup required a discontinuation of capivasertib/placebo only due to AEs; drug eruption was the only AE leading to discontinuation in more than one patient (*n* = 2; 5.4%).

## Discussion

In this descriptive analysis of the Japan subgroup of patients from CAPItello-291, a clinically meaningful improvement in PFS was observed with the addition of capivasertib to fulvestrant, including those patients with *PIK3CA/AKT1/PTEN*-altered tumors. The observed improvement in PFS in the Japan subgroup is in line with findings from the PFS analysis in the global CAPItello-291 population favoring capivasertib–fulvestrant [16]. Similar to findings from the global CAPItello-291 population [16], analysis in the Japan subgroup of patients with confirmed *PIK3CA/AKT1/PTEN*-non-altered tumors suggests that the addition of capivasertib to fulvestrant exerts a PFS benefit regardless of the presence of *PIK3CA/AKT1/PTEN* tumor alterations, although hazard ratios suggest benefit is less pronounced. It should be noted that both the capivasertib–fulvestrant and placebo–fulvestrant arms in the Japan subgroup had longer median PFS versus that observed in the global CAPItello-291 population across the three groups studied (overall population, patients with *PIK3CA/AKT1/PTEN*-altered tumors and patients with *PIK3CA/AKT1/PTEN non*-altered tumors). This is expected given fewer patients from Japan had been treated with prior endocrine therapy or CDK4/6 inhibitors for advanced breast cancer compared with the global CAPItello-291 population; fewer patients from Japan had also received prior chemotherapy. Differences in disease characteristics between the two populations also indicate that the Japan subgroup of patients in CAPItello-291 likely represents an advanced breast cancer population with a less aggressive disease biology at baseline than the global CAPItello-291 population; a higher proportion of patients from Japan had ECOG PS 0 and fewer patients had liver metastases. The proportion of patients with ER-positive/PgR-negative disease, which tends to be associated with a poor outcome on endocrine therapy, was also lower in the Japan subgroup than the global population, which may have also contributed to the observed differences in outcome.

The safety profile of capivasertib–fulvestrant in the Japan subgroup was broadly consistent with the global CAPItello-291 population; no new safety concerns specific to patients in Japan were identified. The relatively higher frequency of grade 3 AEs and AEs leading to discontinuation in the Japan subgroup compared to the overall global population was driven by the more frequent reporting of grade 3 rash and drug eruptions. Drug eruption was only reported in the Japan subgroup and, due to the low numbers, it is difficult to conclude with certainty whether the increased frequency of drug eruption was because of increased sensitivity in patients in the Japan subgroup, differences in baseline characteristics or other unknown factors.

The analysis of capivasertib–fulvestrant in the Japan subgroup of patients from CAPItello-291 has some limitations including the small number of patients and the descriptive nature of the analysis, and when interpreting the results, it is important to acknowledge that the Japan subgroup had a higher proportion of patients with a relatively good prognosis compared with the global CAPItello-291 population. While relatively few patients from Japan had received prior CDK4/6 inhibitors for advanced disease, results from the global population showed that treatment with capivasertib–fulvestrant improved outcomes compared to fulvestrant monotherapy regardless of previous exposure to a CDK4/6 inhibitor.

## Conclusion

Outcomes in the Japan subgroup were in line with the findings in the global CAPItello-291 population further supporting the clinical benefit of capivasertib–fulvestrant in treating patients with HR-positive/HER2-negative advanced breast cancer that has progressed on, or after, an endocrine-based regimen, including patients from Japan. Capivasertib–fulvestrant is approved in Japan for the treatment of adult patients with unresectable or recurrent *PIK3CA*, *AKT1* or *PTEN*-altered HR-positive/HER2-negative breast cancer following progression after treatment with endocrine therapy [20], and represents a novel treatment option for this patient population. CAPItello-291 is ongoing.

## Supplementary information

Below is the link to the electronic supplementary material.

Supplementary file1 (DOCX 374 KB)

## Data Availability

Data underlying the findings described in this manuscript may be obtained in accordance with AstraZeneca’s data sharing policy described at https://astrazenecagrouptrials.pharmacm.com/ST/Submission/Disclosure. Data for studies directly listed on Vivli can be requested through Vivli at www.vivli.org. Data for studies not listed on Vivli could be requested through Vivli at https://vivli.org/members/enquiries-about-studies-not-listed-on-the-vivli-platform/. AstraZeneca Vivli member page is also available outlining further details: https://vivli.org/ourmember/astrazeneca/.

## References

[1] Ferlay J, Ervik M, Lam F, Colombet M, Mery L, Piñeros M, et al. Global cancer observatory: cancer today. Lyon, France: International Agency for Research on Cancer. https://gco.iarc.fr/today/en. Accessed 14 May 2024

[2] Heer E, Harper A, Escandor N, Sung H, McCormack V, Fidler-Benaoudia MM. Global burden and trends in premenopausal and postmenopausal breast cancer: a population-based study. Lancet Glob Health. 2020;8(8):e1027–37. 32710860 10.1016/S2214-109X(20)30215-1

[3] Toyoda Y, Tabuchi T, Nakayama T, Hojo S, Yoshioka S, Maeura Y. Past trends and future estimation of annual breast cancer incidence in Osaka, Japan. Asian Pac J Cancer Prev. 2016;17(6):2847–52. 27356700

[4] Tada K, Kumamaru H, Miyata H, Asaga S, Iijima K, Ogo E, et al. Characteristics of female breast cancer in japan: annual report of the National Clinical Database in 2018. Breast Cancer. 2023;30(2):157–66. 36547868 10.1007/s12282-022-01423-4 PMC9950166

[5] Gennari A, André F, Barrios C, Cortés J, De Azambuja E, DeMichele A, et al. ESMO Clinical Practice Guideline for the diagnosis, staging and treatment of patients with metastatic breast cancer. Ann Oncol. 2021;32(12):1475–95. 34678411 10.1016/j.annonc.2021.09.019

[6] Cardoso F, Paluch-Shimon S, Schumacher-Wulf E, Matos L, Gelmon K, Aapro M, et al. 6th and 7th International Consensus Guidelines for the management of advanced breast cancer (ABC Guidelines 6 and 7). Breast. 2024;76: 103756. 38896983 10.1016/j.breast.2024.103756 PMC11231614

[7] Referenced with permission from the NCCN Clinical Practice Guidelines in Oncology (NCCN Guidelines^®^) for Breast Cancer Version 4.2024. © National Comprehensive Cancer Network, Inc. 2024. All rights reserved. Accessed August 29, 2024. To view the most recent and complete version of the guideline, go online to NCCN.org. NCCN makes no warranties of any kind whatsoever regarding their content, use or application and disclaims any responsibility for their application or use in any way.

[8] Terada M, Ito A, Kikawa Y, Koizumi K, Naito Y, Shimoi T, et al. The Japanese Breast Cancer Society Clinical Practice Guidelines for systemic treatment of breast cancer, 2022 edition. Breast Cancer. 2023;30(6):872–84. 10.1007/s12282-023-01505-x PMC10587293 37804479

[9] Millis SZ, Ikeda S, Reddy S, Gatalica Z, Kurzrock R. Landscape of phosphatidylinositol-3-kinase pathway alterations across 19 784 diverse solid tumors. JAMA Oncol. 2016;2(12):1565–73. 27388585 10.1001/jamaoncol.2016.0891

[10] Toss A, Piacentini F, Cortesi L, Artuso L, Bernardis I, Parenti S, et al. Genomic alterations at the basis of treatment resistance in metastatic breast cancer: clinical applications. Oncotarget. 2018;9(60):31606–19. 30167082 10.18632/oncotarget.25810 PMC6114971

[11] Ma CX, Reinert T, Chmielewska I, Ellis MJ. Mechanisms of aromatase inhibitor resistance. Nat Rev Cancer. 2015;15(5):261–75. 25907219 10.1038/nrc3920

[12] Abu-Khalaf MM, Alex Hodge K, Hatzis C, Baldelli E, El Gazzah E, Valdes F, et al. AKT/mTOR signaling modulates resistance to endocrine therapy and CDK4/6 inhibition in metastatic breast cancers. NPJ Precis Oncol. 2023;7(1):18. 36797347 10.1038/s41698-023-00360-5 PMC9935518

[13] O’Leary B, Cutts RJ, Liu Y, Hrebien S, Huang X, Fenwick K, et al. The genetic landscape and clonal evolution of breast cancer resistance to palbociclib plus fulvestrant in the PALOMA-3 trial. Cancer Discov. 2018;8(11):1390–403. 30206110 10.1158/2159-8290.CD-18-0264 PMC6368247

[14] Frogne T, Jepsen JS, Larsen SS, Fog CK, Brockdorff BL, Lykkesfeldt AE. Antiestrogen-resistant human breast cancer cells require activated protein kinase B/Akt for growth. Endocr Relat Cancer. 2005;12(3):599–614. 16172194 10.1677/erc.1.00946

[15] Davies BR, Greenwood H, Dudley P, Crafter C, Yu DH, Zhang J, et al. Preclinical pharmacology of AZD5363, an inhibitor of AKT: pharmacodynamics, antitumor activity, and correlation of monotherapy activity with genetic background. Mol Cancer Ther. 2012;11(4):873–87. 22294718 10.1158/1535-7163.MCT-11-0824-T

[16] Turner NC, Oliveira M, Howell SJ, Dalenc F, Cortes J, Gomez Moreno HL, et al. Capivasertib in hormone receptor-positive advanced breast cancer. N Engl J Med. 2023;388(22):2058–70. 37256976 10.1056/NEJMoa2214131 PMC11335038

[17] Rugo HS, Oliveira M, Howell SJ, Dalenc F, Cortes J, Gomez HL, et al. Capivasertib and fulvestrant for patients with hormone receptor-positive advanced breast cancer: characterization, time course, and management of frequent adverse events from the phase III CAPItello-291 study. ESMO Open. 2024;9(9): 103697. 39241495 10.1016/j.esmoop.2024.103697 PMC11406080

[18] Oliveira M, Rugo HS, Howell SJ, Dalenc F, Cortes J, Gomez HL, et al. Capivasertib and fulvestrant for patients with hormone receptor-positive, HER2-negative advanced breast cancer (CAPItello-291): patient-reported outcomes from a phase 3, randomised, double-blind, placebo-controlled trial. Lancet Oncol. 2024;25(9):1231–44. 39214106 10.1016/S1470-2045(24)00373-5

[19] US Food and Drug Administration. FDA approves capivasertib with fulvestrant for breast cancer. 2023. https://www.fda.gov/drugs/resources-information-approved-drugs/fda-approves-capivasertib-fulvestrant-breast-cancer. Accessed 27 Feb 2024

[20] Pharmaceuticals and Medical Devices Agency. Truqap^®^Tablets 160 mg, Truqap^®^Tablets 200 mg (package insert, Japan) 2024. https://www.info.pmda.go.jp/go/pack/42910G4F1023_1_02/.

[21] Burstein HJ, DeMichele A, Fallowfield L, Somerfield MR, Henry NL, Biomarker Testing and Endocrine and Targeted Therapy in Metastatic Breast Cancer Expert Panels, et al. Endocrine and targeted therapy for hormone receptor-positive, human epidermal growth factor receptor 2-negative metastatic breast cancer—capivasertib-fulvestrant: ASCO rapid recommendation update. J Clin Oncol. 2024;42(12):1450–3. 38478799 10.1200/JCO.24.00248

