# Supplementary material for: Capivasertib and fulvestrant for patients with HR-positive/HER2-negative advanced breast cancer: analysis of the subgroup of patients from Japan in the phase 3 CAPItello-291 trial
Source: Breast Cancer. 2024 Oct 8;32(1):132–43. doi: 10.1007/s12282-024-01640-z (PMC11717841; doi:10.1007/s12282-024-01640-z)
Supplement: Supplementary file 1 — Supplementary file1 (DOCX 374 KB) [file 12282_2024_1640_MOESM1_ESM.docx]

## Supplementary Appendix

This appendix has been provided by the authors to give readers additional information about their work.

**Supplemental Table 1 *PIK3CA/AKT1/PTEN* tumor alteration status by next-generation sequencing (Japan subgroup and global CAPItello-291 population)**

|  | | Japan subgroup | | Global CAPItello-291 population | |
| --- | --- | --- | --- | --- | --- |
|  | | Capivasertib-fulvestrant (n=37) | Placebo-fulvestrant  (n=41) | Capivasertib-fulvestrant (n=355) | Placebo-fulvestrant  (n=353) |
| Any alteration, n (%) | | 19 (51.4) | 19 (46.3) | 155 (43.7) | 134 (38.0) |
| *PIK3CA* | *PIK3CA* only | 10 (27.0) | 11 (26.8) | 110 (31.0) | 92 (26.1) |
|  | *PIK3CA* and *AKT1* | 0 | 0 | 2 (0.6) | 2 (0.6) |
|  | *PIK3CA* and *PTEN* | 0 | 1 (2.4) | 4 (1.1) | 9 (2.5) |
| *AKT1* only |  | 6 (16.2) | 5 (12.2) | 18 (5.1) | 15 (4.2) |
| *PTEN* only |  | 3 (8.1) | 2 (4.9) | 21 (5.9) | 16 (4.5) |
| Non-altered, n (%) | | 18 (48.6) | 22 (53.7) | 200 (56.3) | 219 (62.0) |
| Alteration not detected |  | 14 (37.8) | 20 (48.8) | 142 (40.0) | 171 (48.4) |
| Unknown |  | 4 (10.8) | 2 (4.9) | 58 (16.3) | 48 (13.6) |
|  | No sample available | 0 | 0 | 10 (2.8) | 4 (1.1) |
|  | Pre-analytical failure | 3 (8.1) | 2 (4.9) | 39 (11.0) | 34 (9.6) |
|  | Post-analytical failure | 1 (2.7) | 0 | 9 (2.5) | 10 (2.8) |

*AKT1*, Akt serine/threonine kinase 1; *PIK3CA*, catalytic subunit of phosphatidylinositol-3-kinase; *PTEN*, phosphatase and tensin homologue.

**Supplemental Table 2 Summary of safety (safety population)**

|  | All patients | | | |
| --- | --- | --- | --- | --- |
|  | **Japan subgroup** | | **Global  CAPItello-291 population** | |
| n (%) | Capivasertib-fulvestrant (n=37) | Placebo-fulvestrant  (n=41) | Capivasertib-fulvestrant (n=355) | Placebo-fulvestrant  (n=350) |
| Any AE | 37 (100) | 34 (82.9) | 343 (96.9) | 288 (82.3) |
| Any serious AE | 5 (13.5) | 2 (4.9) | 57 (16.1) | 28 (8.0) |
| Any AE leading to death | 0 | 0 | 4 (1.1) | 1 (0.3) |
| Any AE leading to discontinuation | 9 (24.3) | 0 | 46 (13.0) | 8 (2.3) |
| Discontinuation of capivasertib/placebo only | 7 (18.9) | 0 | 33 (9.3) | 2 (0.6) |
| Discontinuation of both capivasertib/placebo and fulvestrant | 2 (5.4) | 0 | 13 (3.7) | 6 (1.7) |
| Any AE leading to dose interruption of capivasertib/placebo only | 21 (56.8) | 5 (12.2) | 124 (34.9) | 36 (10.3) |
| Any AE leading to dose reduction of capivasertib/placebo only | 10 (27.0) | 2 (4.9) | 70 (19.7) | 6 (1.7) |

AE, adverse event.

### Supplemental Figure 1 CONSORT diagram


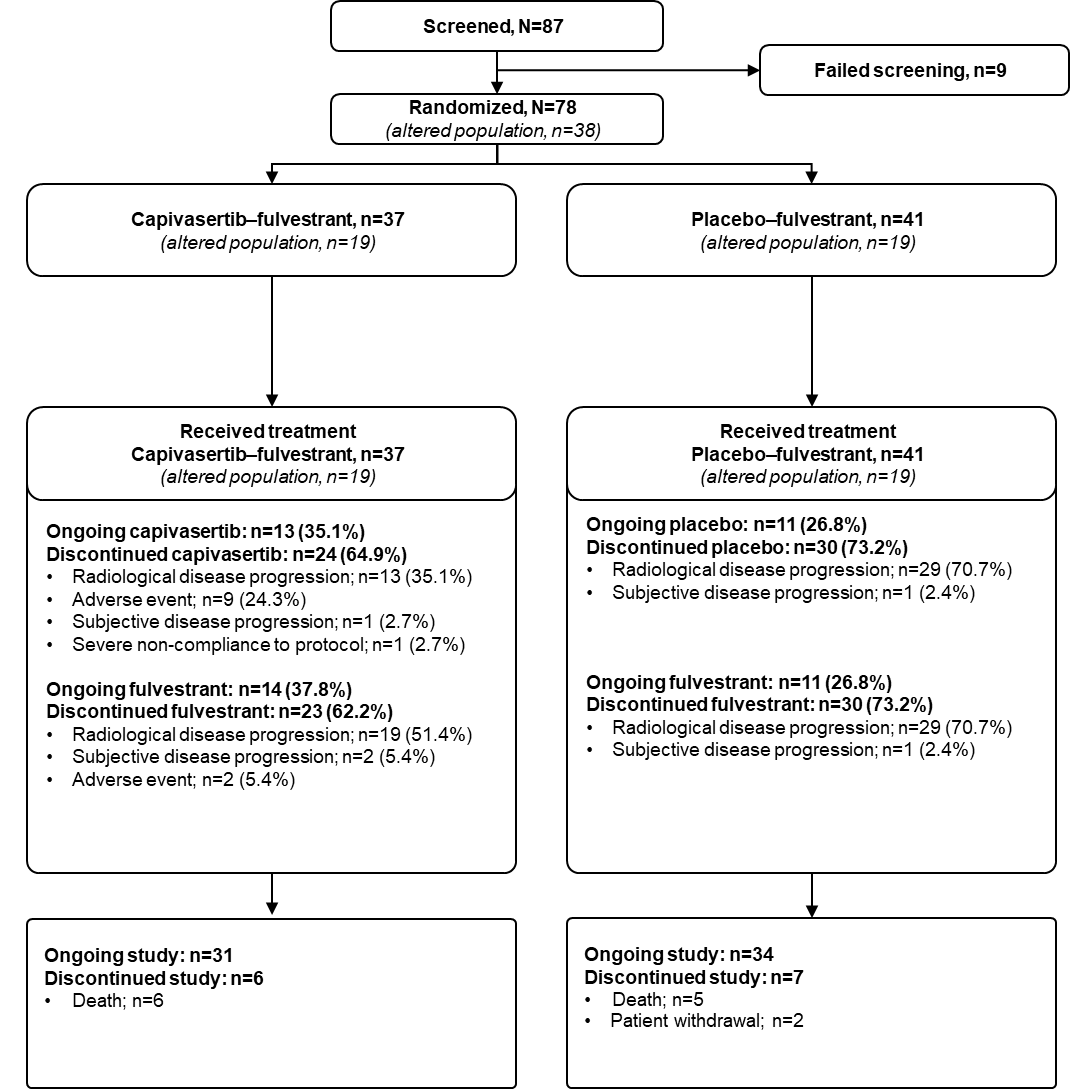


### Supplemental Figure 2 Investigator-assessed PFS in patients with *PIK3CA/AKT1/PTEN-*non-altered tumors^a^ in a) the Japan subgroup, and b) the global CAPItello-291 population

A


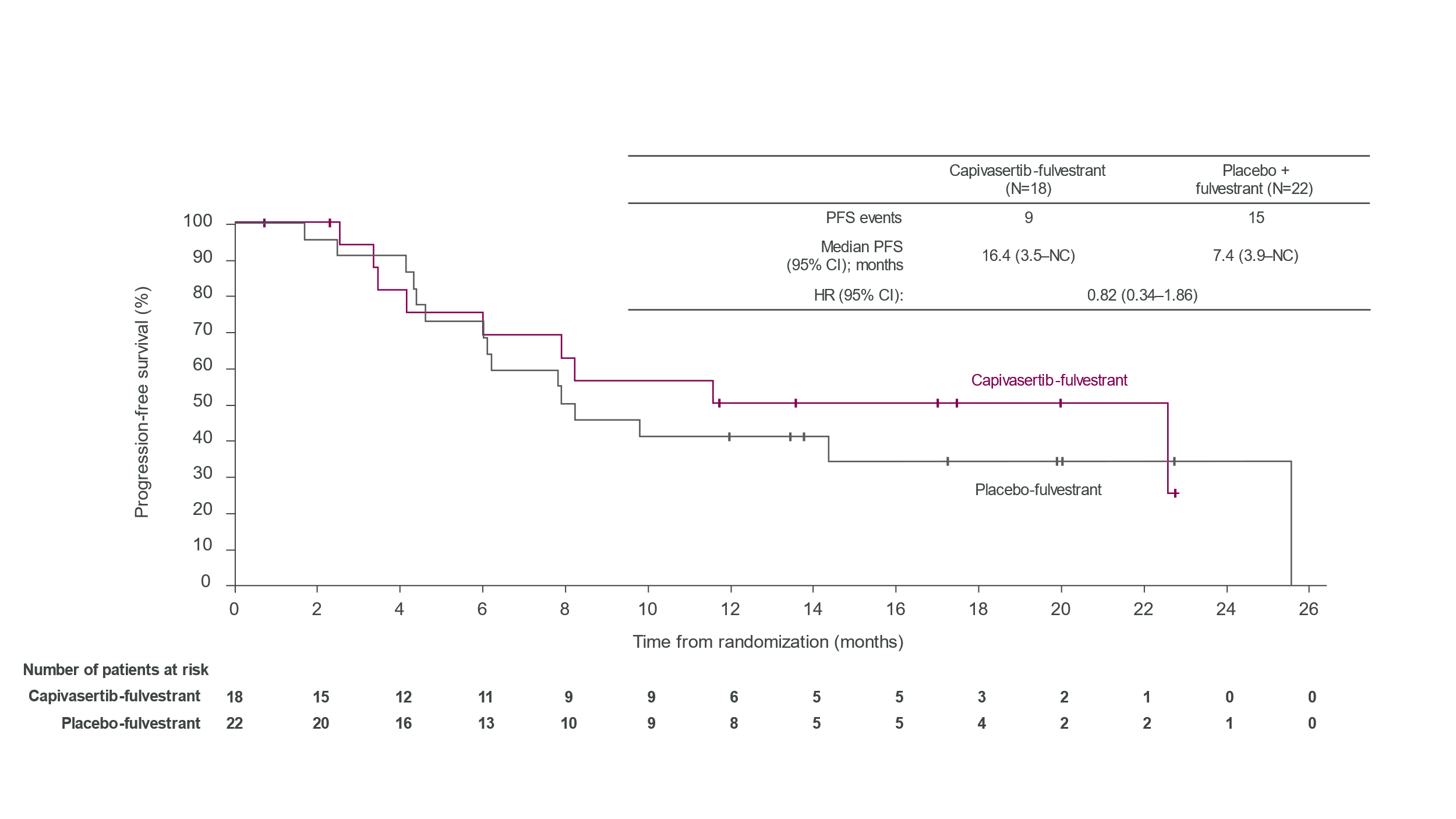


B


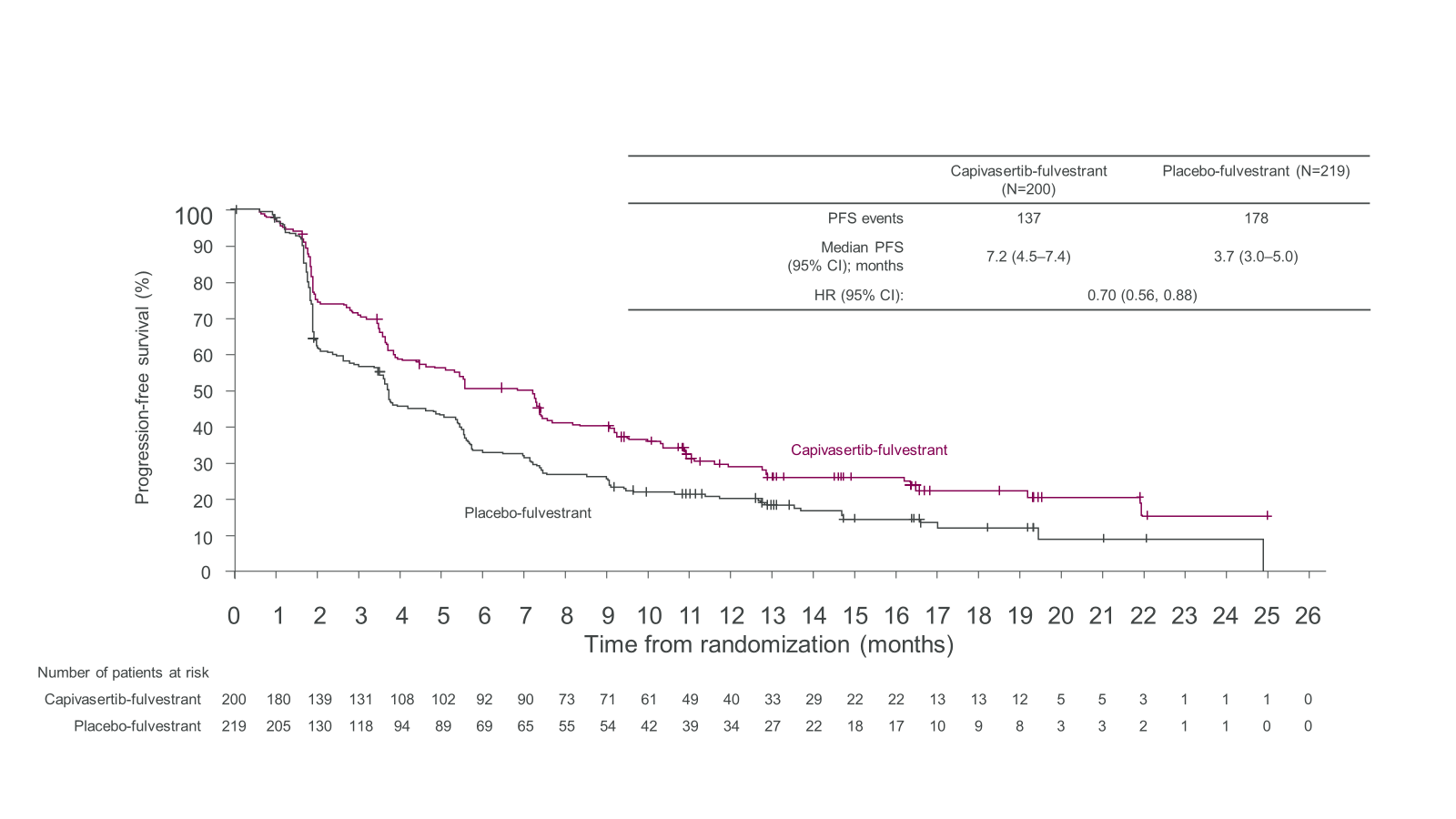


The HR was estimated in the Japan subgroup using an unstratified Cox proportional hazards model, and in the global CAPItello-291 population using the Cox proportional hazards model stratified according to the presence or absence of liver metastases and previous CDK4/6 inhibitor use. Tick marks indicate censored data.

^a^Includes unknowns (patients with no valid next-generation sequencing results).

Panel b From New England Journal of Medicine. Turner NC, Oliveira M, Howell SJ, Dalenc F, Cortes J, Gomez Moreno HL, et al. Capivasertib in hormone receptor-positive advanced breast cancer. Volume 388., Page No 2064. Copyright © (2023) Massachusetts Medical Society. Reprinted with permission.

*AKT1*, Akt serine/threonine kinase 1; CI, confidence interval; HR, hazard ratio; PFS, progression-free survival; *PIK3CA*, catalytic subunit of phosphatidylinositol-3-kinase; *PTEN*, phosphatase and tensin homologue.
